# Ion transporter gene expression is linked to the thermal sensitivity of calcification in the reef coral *Stylophora pistillata*

**DOI:** 10.1038/s41598-019-54814-7

**Published:** 2019-12-10

**Authors:** C. Bernardet, E. Tambutté, N. Techer, S. Tambutté, A. A. Venn

**Affiliations:** 1 Centre Scientifique de Monaco, Marine Biology Department, 8 Quai Antoine 1er, Monaco, 98000 Monaco; 2 Sorbonne Université, Collège Doctoral, F-75005 Paris, France

**Keywords:** Ecophysiology, Marine biology

## Abstract

Coral calcification underpins biodiverse reef ecosystems, but the physiology underlying the thermal sensitivity of corals to changing seawater temperatures remains unclear. Furthermore, light is also a key factor in modulating calcification rates, but a mechanistic understanding of how light interacts with temperature to affect coral calcification is lacking. Here, we characterized the thermal performance curve (TPC) of calcification of the wide-spread, model coral species *Stylophora pistillata*, and used gene expression analysis to investigate the role of ion transport mechanisms in thermally-driven declines in day and nighttime calcification. Focusing on genes linked to transport of dissolved inorganic carbon (DIC), calcium and H^+^, our study reveals a high degree of coherence between physiological responses (e.g. calcification and respiration) with distinct gene expression patterns to the different temperatures in day and night conditions. At low temperatures, calcification and gene expression linked to DIC transport processes were downregulated, but showed little response to light. By contrast, at elevated temperature, light had a positive effect on calcification and stimulated a more functionally diverse gene expression response of ion transporters. Overall, our findings highlight the role of mechanisms linked to DIC, calcium and H^+^ transport in the thermal sensitivity of coral calcification and how this sensitivity is influenced by light.

## Introduction

Increases in seawater temperature driven by global warming are a first-order threat facing coral reef ecosystems worldwide. Together with aspects of global change including ocean acidification and declining water quality, changing seawater temperatures challenge several aspects of reef coral physiology, with severe ramifications at the ecosystem level^1–4^.

Among the impacts of temperature on reef corals, coral bleaching is the most widely studied. In this process, elevated temperatures provoke the breakdown of the symbiosis between corals and their photosynthetic dinoflagellate algae (family Symbiodiniaceae) following cellular stress events occurring in both the coral host and symbiont^5–7^. Additionally, temperature change may impact rates at which corals build their skeletons by calcification^8^. Studies on coral skeletons from several geographical locations indicate that this has become a worldwide problem in the context of climate change in recent decades^9–12^.

The threat of changing seawater temperatures has led to intense research into the mechanisms that determine the thermal sensitivity of corals e.g.^13–18^. By deciphering how and why corals respond to temperature at molecular and cellular scales this work provides insight into their vulnerability and their capacity to acclimatize and tolerate periods of elevated temperature^19^. Ultimately, such information is also important for initiatives that are actively exploring interventions to improve the physiological resilience of corals to future environmental change, including human-assisted evolution and synthetic biology^20,21^.

Much of the previous research into the cellular mechanisms underlying the thermal sensitivity of corals has focused on coral bleaching e.g.^14,16,18^, with less mechanistic work having addressed the thermal sensitivity of coral calcification. Certain studies on bleaching mechanisms have examined gene expression profiles against a backdrop of physiological parameters linked to the bleaching response, such as decreases in photosynthetic efficiency and declines in symbiont numbers^18^. Few studies of this type have been carried out directly on calcification, although there are, however, gene expression studies that have focused on combined thermal and ocean acidification stress^22,23^. Nevertheless, one important theme emerging from previous gene expression work on thermal stress on corals is that disruption to ion transport processes may underlie the response of coral calcification to temperature changes^17,24^. Indeed, a review of gene expression biomarkers in corals highlights certain ion transport genes as heat-stress markers^25^. However, the links between ion transport mechanisms and the thermal sensitivity of corals has never been directly investigated.

Even if the coral calcification mechanism itself is not fully understood, the ion transport for calcification is known to be central to the process, particularly movement of Ca^2+^, H^+^ and dissolved inorganic carbon (DIC)^26–28^. The coral skeleton is separated from the surrounding environment by the coral tissues, which are comprised of oral and aboral epithelia separated by a lumen called the coelenteron. These epithelia regulate the ionic composition of the extracellular calcifying medium (ECM), which lies between the aboral layer’s calcifying cells (calicoblastic epithelium) and the skeleton^28^. Research into the ionic composition of the ECM has shown that corals regulate Ca^2+^ and dissolved inorganic carbon concentrations, as well as pH in the ECM to levels above that of the exterior seawater^29–34^. The effect of increasing these parameters is to elevate aragonite saturation state which promotes growth of the skeleton^34,35^. The difference in concentration between [Ca^2+^], [H^+^], and [DIC] in the ECM and the surrounding seawater implies that these ions are regulated by primary and secondary active transport mechanisms. Research has sought to characterize mechanisms of ion transport to the ECM and the skeleton, and to identify and localize some of the transporters involved^28,36^. This has included studies of Ca^2+^ and DIC transport using radiotracer experiments^37,38^ and the localization of a calcium channel, calcium ATPase, various carbonic anhydrases and bicarbonate/chloride exchangers^39–42^. Additionally, recent research is beginning to elucidate other steps in calcification including the formation of calcium carbonate particles in intracellular vesicles and the role of organic matrix proteins in catalyzing crystal nucleation and forming a template for skeletal growth^43–48^. While these latter studies are evolving our understanding of coral calcification, it is clear that ion transport remains a fundamental part of the overall mechanism.

Ion transport involved in calcification must be viewed in context with other aspects of coral metabolism including respiration and photosynthesis, as these parameters are intrinsically linked^49^. Indeed, it has long been known that photosynthesis can augment calcification rates in a process known as light-enhanced calcification^40,49–51^. The mechanisms underlying the links are uncertain, but previous research suggests that photosynthesis may increase calcification rates by driving an elevation in pH in the coelenteron which facilitates pH increase in the ECM^52^. Moreover, respiration provides a significant source of DIC for the calcification reaction^38^. Also, it has been proposed that respiration supplies ATP for active transport mechanisms linked to calcification^52^, and that photosynthesis leads to the provision of oxygen and carbon compounds from coral symbionts that may increase respiration rates^53^. It is clear therefore, that investigations into ion transport mechanisms linked to calcification also need to take photosynthesis and respiration into account.

The overarching aim of the current study was to improve mechanistic understanding of how temperature causes declines in coral calcification. We pursued this aim by focusing on the expression of a selected group of genes with functions in the transport and regulation of Ca^2+^, H^+^ and DIC selected from the genome of our model species *Stylophora pistillata*^54,55^. These genes were chosen from genes described in the existing literature with putative links to calcification, respiration or photosynthesis (see Supplementary Information Table 1 for references). Before gene expression analysis, we first characterized the thermal performance curves (TPCs) of coral calcification, photosynthesis and respiration rates in day and night time conditions over a range of 17–33 °C (one-week exposure). Data from the TPCs allowed us to identify temperatures where calcification rates decreased significantly in distinctly different physiological scenarios (17 and 32 °C relative to 25 °C). Gene expression analysis was then carried out after one week’s exposure at these target temperatures.

## Material and Methods

### Biological materials and experimental design

Experiments were conducted with the tropical symbiotic scleractinian species *Stylophora pistillata* (Esper, 1797) which is kept in long-term culture at the Centre Scientifique de Monaco. Branch tips of 5 cm in length were cut from three mother colonies of a single genotype and kept for three weeks in a 120-L tank supplied with Mediterranean seawater at a temperature of 25 °C, salinity 38, pH_T_ 7.94 ± 0.02 and irradiance of 230 µmol photons m^−2^s^−1^ on a 12:12 photoperiod. Corals were fed daily with frozen rotifers and twice a week with live artemia nauplii.

After three weeks, samples were transferred to 30-L experimental tanks for week-long temperature experiments to characterize TPCs and gene expression. Apart from temperature, conditions in these tanks matched those described above for the 120-L tank.

The experiments were carried out in two steps. In the first step, one set of samples were used to characterize the TPC of physiological parameters after 1-week exposure to the following temperatures: 17, 19, 21, 23, 25, 27, 29, 31, 32 and 33 °C. Measurements of calcification, photosynthesis and respiration rates were determined under each treatment condition. Night time measurements were conducted in darkness at the end of the night before aquarium lights were switched on. Day time measurements were carried out in the light at midday.

Once we had identified temperatures at which calcification rate was significantly decreased relative to 25 °C from the TPC, another set of samples was used to characterize the expression of genes linked to ion transport in the second step of the investigation. This time, week-long exposures were only carried out at three temperatures: 17, 25 and 32 °C. Samples at 33 °C were not included in gene expression analysis due to the severe bleaching they experienced. Night and day sampling occurred at the same times used in characterization of the TPC in the first part of the study.

### Calcification rate

Net calcification rate was measured by the alkalinity anomaly method^56^. Briefly, 50-ml closed beakers containing coral samples or blank seawater samples agitated with a magnetic stirrer were kept at the target temperature with a water bath for 30 min. For day-time measurements light was provided at 230 µmol photons m^−2^s^−1^. At the end of the incubation, the water in the beakers was collected to perform titrations as described in Tambutté *et al*. (2015). Titrations were performed in triplicate on each sample to check for measurement precision. Typically, depletion in alkalinity due to calcification was more than 10 fold greater than measurement precision. Calcification rates were calculated from the difference in total alkalinity (TA) between seawater in beakers containing samples and blanks, correcting for sample displacement volume. Calcification rates were determined in eighteen samples at 25 °C and six samples for all other temperatures. Values were normalized to the surface area of the samples.

### Photosynthesis and respiration rates

Samples were placed in 50-ml closed beakers under agitation in a water bath set at the desired temperature and the concentration of oxygen was measured each 15 seconds for 20 min by an oxygen optode sensor system (oxy-4 mini, PreSens, Regensburg, Germany). Data were recorded with OXY4v2_09 software (PreSens). Before each measurement, the oxygen sensor was calibrated with a saturated solution of sodium sulfite (0% oxygen) and oxygen-saturated seawater (100%) using seawater from a beaker with O_2_ bubbling.

Night respiration was measured at the end of the night period before aquarium lights switched on, while net photosynthesis and day respiration measurements were conducted at midday. Light was provided at 230 µmol photons m^−2^s^−1^. Changes in oxygen concentration with time were determined at all temperatures in light and darkness, except 27 °C in the light where a technical problem prevented acquisition of data. Photosynthesis and respiration rates were determined in eighteen samples at 25 °C and six samples for all other temperatures. Values were normalized by the surface area of the samples.

### Symbiotic dinoflagellate density

Samples were air-picked with dinitrogen in 10 ml of filtered sea water (FSW). The resulting slurry was then centrifuged (at 1500 g for 10 min) and resuspended in 10 ml of MilliQ water for 15 min, centrifuged a second time (at 1500 g for 10 min) and resuspended in 5 ml of FSW. A 15 µl aliquot was deposited on a Mallassez cell for counting under a Leica DM750P microscope at X40 magnification. Symbiotic dinoflagellate density was determined in three samples per temperature. Values were normalized by the surface area of the samples

### Microcolony surface area

Colony surface area was measured using the paraffin wax method^57^. Briefly, coral skeletons were coated in paraffin wax by dipping in Paraplast wax (Sigma, France) at 65 °C. Surface area of the specimens was obtained by referring the weight of the paraffin wax coated on the specimen to the standard curve of paraffin wax versus surface area. The standard curve was generated by regressing weight of the paraffin wax to known surface area density blocks^58^.

### RNA extraction

Samples were flash frozen in liquid nitrogen and placed in a −80 °C freezer before RNA extraction. Samples were air-picked with dinitrogen in 600 µl of RLT buffer (Qiagen). Total RNA was then extracted according to the protocol of RNeasy ® Mini Kit (Qiagen). RNA quantification was conducted with a microplate spectrophotometer Epoch (Bioteck) using Gen5 2.03 software. Quality of RNA was also checked by running the samples on an agarose gel 1% which was then observed under UV-exposure with Fusion Fx7.

### Reverse transcription and real-time PCR

Reverse transcription (RT) was carried out according to Invitrogen’s SuperScript IV Reverse Transcriptase (2000 U/µl) User Guide (Protocol Pub. No. MAN0013443 Rev. A.0), using 1 µg of RNA. The RT was carried out in a PCR machine Eppendorf ® Mastercycler gradient. A first step at 23 °C allowed random hexamers to bind RNA strands, a second step at 50 °C allowed the reverse transcription and a third step at 80 °C ended the reaction. A control PCR was then conducted to check RT products.

Real-time PCR (qPCR) was used to analyse the expression of a suite of genes linked to ion transport (Table Supplementary Material 1). These included 17 genes coding for: three plasma membrane calcium ATPases (PMCA 1, 2 and 3), four carbonic anhydrases (SpisCA 1 to 4), bicarbonate transporters SpisSLC4 β, γ, δ and ε and SpisSLC26 α, β and γ, calcium channel, V-ATPase and a Na^+^-H^+^ exchanger (NHE) (Primer sequences are given in Supplementary Material Table 1).

qPCR was conducted in 96-wells plates with the qPCR machine QuantStudio 3 (Applied Biosystems) using PowerUp^TM^ SYBR^TM^ Green Master Mix. Samples were analysed in triplicate using 1 ng of cDNA. Non template controls were included on each reaction plate and an internal standard was also included to test for amplification variability between runs. Primer efficiency and gene expression was analyzed with qBase + software (Biogazelle)^59^. Gene expression was normalized to the geometric mean of expression of two reference genes (36B4 and L22) after these references genes were determined to have acceptably low M values and coefficient of variations^59^. Gene expression was determined in 6 samples per treatment.

### Statistical analyses

Statistical analysis was performed using R v3.5.2 software. Data were checked for normality with a normal probability plot of residuals. Calcification and respiration data were analyzed by two-way ANOVA. Photosynthetic rate was analyzed by one-way ANOVA. For calcification and respiration post-hoc analysis was carried out by pairwise t tests. For photosynthesis, a Student-Newman-Keuls test was performed. Polynomial regressions were also carried out for calcification, respiration and photosynthesis data.

For gene expression analysis, we first conducted discriminant analysis to explore global patterns in the data. We followed this with analysis of the gene expression by three-way ANOVA. The factors were as follows: Temperature (17, 25 and 32 °C), time of day (daytime and nighttime) and gene identity (the 17 genes analyzed). Post-hoc analysis was carried out by pairwise t tests to discriminate the genes which were expressed differently at 17 °C and 32 °C compared to 25 °C, both during night and day.

## Results

### Thermal performance curves

**Figure 1:**
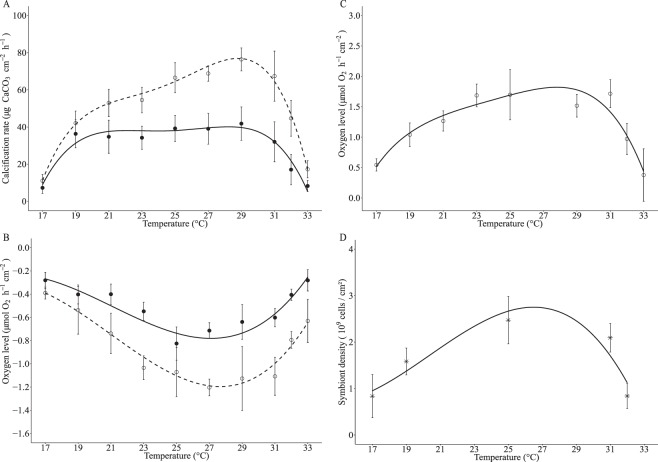
Physiological parameters at different temperatures (mean ± SD) (n = 18 microcolonies for 25 °C and n = 6 microcolonies for other temperatures). Closed circles (●) represent night and open dots (○) day measurements. Polynomial regressions indicate trend of thermal performance curves (TPCs). (**A**) Calcification rate (night P < 0.001, R² = 0.71 and day P < 0.001, R² = 0.88), (**B**) Respiration rate (night P < 0.001, R² = 0.76 and day P < 0.001, R² = 0.69), (**C**) Net photosynthesis rate (P < 0.001, R² = 0.68) and (**D**) Symbiont density (P = 0.48, R² = 0.85).

The response of calcification and respiration rates to temperature was assessed across a range of 17 °C to 33 °C during the night and the day (Fig. 1A,B). Photosynthetic rate was also measured during the day (Fig. 1C) and dinoflagellate symbiont densities were determined at 17, 19, 25, 32 and 33 °C (Fig. 1D). All parameters were analyzed by regression analysis to reveal the trend of the data (Fig. 1) and by two or one-way ANOVA (Supplementary Material Table 2–4).

Physiological parameters generally displayed typical TPCs, with rates increasing with temperature until an optimum and declining at higher temperatures. During the night, calcification and respiration rates declined to similar extents at lower and elevated temperatures relative to 25 °C (Fig. 1A,B, Supplementary Information Tables 2 and 3) and were significantly correlated (Supplementary Information Fig. 1). Likewise, during the day, calcification, respiration and photosynthesis declined towards the ends of the TPC and were significantly correlated (Supplementary Information Fig. 1) (Supplementary Material Tables 2–4). Analysis by ANOVA (Supplementary Information Tables 2–4) indicated that calcification, respiration and photosynthesis rates were all significantly decreased at 17, 32 and 33 °C with respect to 25 °C in both day and night conditions.

There was a significant interaction of day/nighttime with temperature for both calcification and respiration (Supplementary Information Tables 2–4). Indeed, day time calcification, respiration and photosynthesis were significantly elevated at higher temperatures (i.e. 32 °C) relative to lower temperatures (i.e. 17 °C) (Fig. 1A–C). By contrast, nighttime calcification and respiration rates were at similar levels at either end of the TPC.

Measurements of symbiotic dinoflagellate density showed that symbiont numbers significantly changed across the temperature range observed here (Fig. 1D). Symbiont counts of samples taken at 17 °C and 32 °C were significantly reduced to similar levels with respect to 25 °C, indicating these samples had undergone similar levels of bleaching during these temperature treatments (Fig. 1D). Coral colonies at 33 °C were noticeably paler than all other colonies and therefore likely to have been severely depleted in symbiont numbers and, as such, these samples were not analyzed.

Normalization of mean photosynthetic rates by symbiont densities (Supplementary Information Fig. 2), indicated that photosynthesis *per se* was not negatively affected by higher and lower temperatures. In fact, higher temperatures (32 °C) increased photosynthesis at the level of the symbiont. This indicates that declines in photosynthesis at the level of the colony (i.e. normalized by surface area) were mainly driven by loss of symbionts, but the increased rates of photosynthesis at 32 °C relative to 17 °C may have occurred through a positive effect of temperature on the photosynthetic reactions.

In summary, characterization of the thermal performance curve allowed us to target 17 °C and 32 °C as low and high temperatures at which coral calcification was significantly decreased relative to 25 °C. These temperatures were selected for gene expression analysis. Samples from 33 °C were not included in the gene expression analysis due to severe bleaching.

### Gene expression

**Figure 2:**
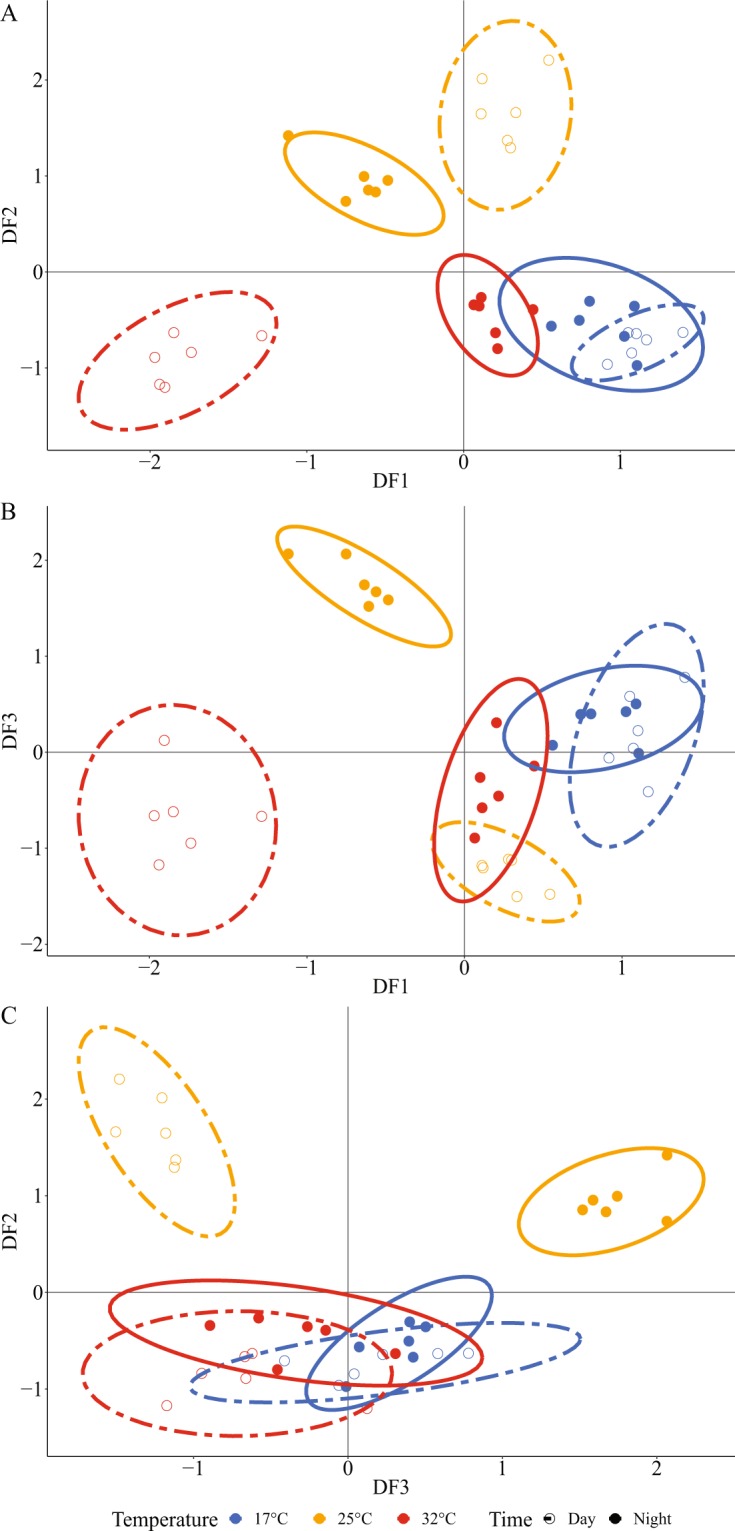
Discriminant analysis on the gene expression of 17 ion transport genes in *S. pistillata*. Each dot represents the gene expression profiles for each sample, and ellipses show the clusters. Blue denotes samples at 17 °C, yellow at 25 °C, and red at 32 °C. Closed dots (●) represent night individuals and open dots (○) represent day individuals. A, B and C show plots of Discriminant Factors (DF). DF1 explains 24% of variation, DF2 explains 24% and DF3 explains 22%. One-way ANOVAs carried out on each of the three axis of the discriminant analysis showed statistical differences between clusters (P < 0.05).

Real-time qPCR analysis was carried out on coral samples in the 17, 25 and 32 °C treatments. The expression of a panel of 17 genes was analyzed, chosen for their roles in DIC, H^+^ and Ca^2+^ transport and regulation, and existing literature that suggests links to calcification, respiration or photosynthesis (Table Supplementary Information 1). First we carried out discriminant analysis of gene expression data which grouped the expression profiles of individual samples into distinct clusters according to temperature (Fig. 2, Supplementary Information Tables 5 and 6). 70% of the variation of the gene expression profiles of the samples was explained by the first three axes. Both 17 °C and 32 °C were separated from the groups at 25 °C. At 25 °C, night and day groups were consistently separated. In contrast, the expression profiles of colonies during night and day at 17 °C always grouped together. Night and day groups at 32 °C overlapped in both DF2 (Discriminant Factor 2) and DF3 axis, but were clearly separated into night and day clusters at 32 °C on the DF1 axis. Samples from night time 32 °C grouped close to the 17 °C day and night groups on all three axes (Fig. 2).

**Figure 3:**
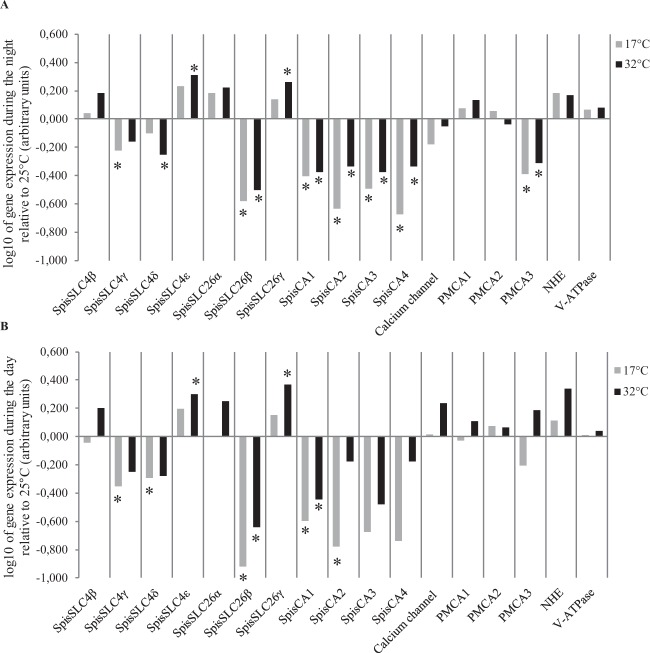
Mean gene expression (n = 6) plotted relative to 25 °C (log10 transformation): (**A**) during the night, and (**B**) during the day. Grey bars correspond to gene expression at 17 °C and black bars at 32 °C. Negative values indicate downregulation and positive values upregulation. Asterisks indicate statistical difference (p < 0.05) with gene expression at 25 °C. Standard deviation error bars and means (not log transformed) are given in the supplementary material.

**Figure 4:**
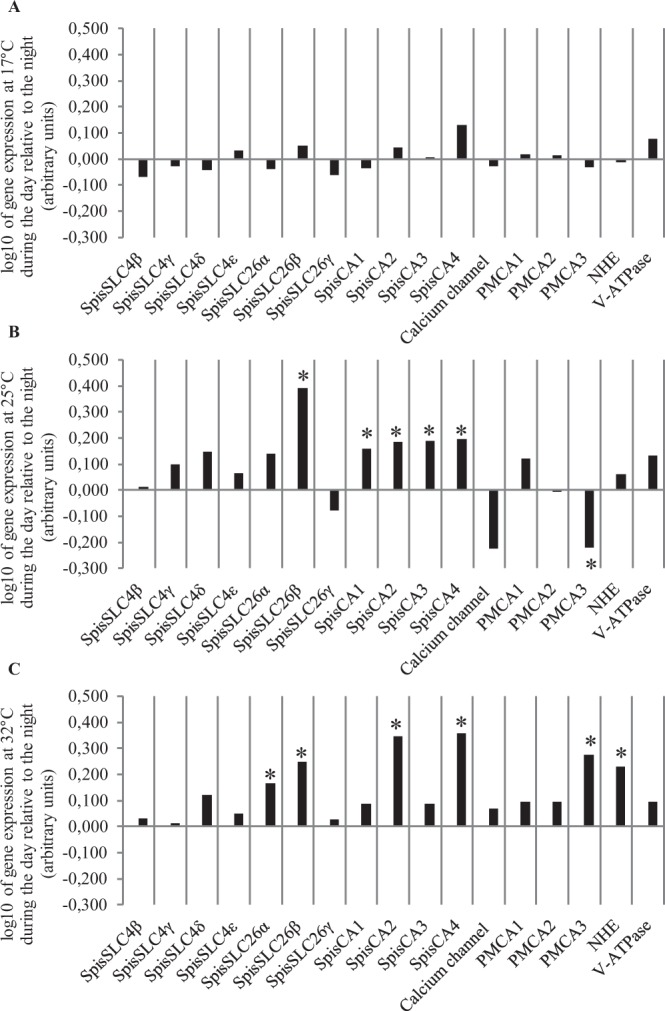
Mean daytime gene expression (n = 6) plotted relative to the night (log10 transformation): (**A**) 17 °C, (**B**) 25 °C, and (**C**) 32 °C. Asterisks indicate statistical difference (p < 0.05) with night time conditions. Negative values indicate downregulation and positive values upregulation. Standard deviation error bars and means (not log transformed) are given in the supplementary material.

Following discriminant analysis, we performed three-way ANOVA which identified significant effects of night/day, temperature and gene on the expression profile of the coral samples (ANOVA results given in Supplementary Information Table 7). In Figs. 3 and 4 we plot gene expression relative to 25 °C and to nighttime expression respectively. Significant interactions were identified between the night/day, temperature and gene, indicating that the genes responded differently during night and day and at different temperatures (Supplementary Material Table 7). Post-hoc analysis identified the expression of which gene responded significantly in the different treatments using an alpha of 0.05 as the statistical cut-off (Tables 1 and 2). In Table 1, we also indicate responses that fell within a cutoff off of p < 0.1 in order to indicate the trend of the response.

**Table 1:** Summary of statistically significant differences in gene expression at 17 °C and 32 °C compared to 25 °C during night and day (Fig. 3) determined by three-way ANOVA and posthoc analysis.

Gene	NIGHT		DAY		Function	
	17 °C	32 °C	17 °C	32 °C		
SpisSLC4ε		↗		↗	DIC transport	
SpisSLC4γ	↘		↘	↘		
SpisSLC26β	↘	↘	↘	↘		
SpisCA1	↘	↘	↘	↘		
SpisCA3	↘	↘	↘	↘		
SpisCA2	↘	↘	↘	↘		
SpisCA4	↘	↘	↘	↘		
SpisSLC4β	—		—	↗		
SpisSLC26γ	—	↗	—	↗		
SpisSLC26α	—		—	↗		
SpisSLC4δ	—	↘	↘	↘		
Ca^2+^ channel		—	—	↗	Ca^2+^ transport	
PMCA1	—	—	—	—		H^+^ transport
PMCA2	—	—	—	—		
PMCA3	↘	↘	—	↗		
NHE	—	—	—	↗		
V-ATPase	—	—	—	—		

Black arrows represent significant increase or decrease in expression relative to 25 °C (p < 0.05). Grey arrows represent trends relative to 25 °C (i.e. p < 0.1).

**Table 2:** Summary of statistically significant differences in gene expression at 17 °C, 25 °C and 32 °C during the day compared to the night (Fig. 4) determined by three-way ANOVA and posthoc analysis.

Gene	DAY			Function	
	17 °C	25 °C	32 °C		
SpisSLC4ε	—	—	—	DIC transport	
SpisSLC4γ	—	—	—		
SpisSLC26β	—	↗	↗		
SpisCA1	—	↗	—		
SpisCA3	—	↗	—		
SpisCA2	—	↗	↗		
SpisCA4	—	↗	↗		
SpisSLC4β	—	—	—		
SpisSLC26γ	—	—	—		
SpisSLC26α	—	—	↗		
SpisSLC4δ	—	—	—		
Ca^2+^ channel	—	—	—	Ca^2+^ transport	
PMCA1	—	—	—		H^+^ transport
PMCA2	—	—	—		
PMCA3	—	↘	↗		
NHE	—	—	↗		
V-ATPase	—	—	—		

Black arrows represent significant increase or decrease in expression relative to the night (i.e. p < 0.05).

Looking at Fig. 3 and Table 1 which compares gene expression between 17 °C and 32 °C with 25 °C, two broad patterns emerged from post-hoc analysis. Firstly, we found that the two ends of the TPC shared a similar core set of genes that were down-regulated in both night and day time conditions. These included the four carbonic anhydrase genes and also the bicarbonate transporters SpisSLC4γ and SpisSLC26β. Secondly, we found that despite similarities between 17 °C and 32 °C, a larger and functionally more diverse set of genes responded at 32 °C in the daytime compared to the 17 °C treatment relative to 25 °C. Indeed, it can be seen from Table 1, that the expression of almost twice as many genes responded significantly at 32 °C in the daytime relative to 17 °C (at the alpha 0.05 cut-off). These genes included additional bicarbonate transporters (e.g. genes belonging to the SpisSLC4 and SpisSLC26 families) and also genes involved in proton transport (NHE) and calcium transport (PMCA3) at 32 °C in day time conditions.

We also compared gene expression between daytime and nighttime across the three temperatures (Fig. 4, Table 2) to highlight the expression of which genes were responsive to daytime conditions. At 17 °C, gene expression did not change significantly in the day or night treatments for any of the genes, however, at 25 °C and 32 °C the expression of a group of genes was responsive to daytime conditions (Table 2). In agreement with Fig. 3 and Table 1, in the 32 °C treatment these genes included SpisSLC26α, NHE, PMCA genes in addition to both SpisCA2 and SpisCA4.

In summary, three-way ANOVA and posthoc analysis reveal firstly the response of the expression of a core group of genes associated with the extremes of the TPC, and secondly a more diverse gene group associated with an interaction between daytime conditions and elevated temperature.

## Discussion

It is widely known that changes in seawater temperature can depress rates of reef coral calcification, but little is understood about how and why. Temperature itself has a direct, abiotic effect on calcification, because precipitation rates of aragonite increase with elevations in temperature^60^. However, the linear relationship of abiotic aragonite precipitation rates with temperature bears little resemblance to the bell-shaped thermal performance curves of calcification observed for *S. pistillata* (see Supplementary Information Fig. 4 for comparison) or similar curves described in previous investigations^8^. This difference underscores the importance of biological factors in shaping the coral calcification response to temperature.

Here, we hypothesized that ion transport mechanisms are among the biological factors that determine how coral calcification responds to temperature. We investigated this hypothesis by targeting our analysis on the expression of a suite of genes with roles in calcium, proton and DIC transport at the extremes of the thermal performance window of *S. pistillata*.

### Coherence of physiology and gene expression

A number of previous studies have investigated the effects of temperature on coral calcification and other physiological parameters by describing thermal performance curves (TPCs) for several coral species^61–63^, including some of the first work conducted on the thermal response of scleractinians^8,64^. Calcification rates in this previous work and the current study have been observed to follow the TPC trend that is characteristic of most physiological processes, in which rates increase with rising temperatures until an optimum, after which they decline^65,66^. Similarly, in the current study photosynthesis and respiration performed in this manner, although loss of symbiotic dinoflagellates appears to have driven the declines in photosynthesis per unit surface area of the coral colony. In any case, when we consider the physiological parameters together it becomes apparent that we observed distinctly different physiological conditions at each end of the TPCs. At low temperatures (17 °C) there was little difference between rates of respiration and calcification between night and daytime conditions, but at elevated temperature (32 °C), daytime calcification and respiration were much greater than in the night. It was under these opposing physiological conditions that we sought to profile the expression of the ion transport genes.

Discriminant analysis of gene expression of ion transporters revealed that the global pattern of gene expression mapped closely with the physiological parameters observed at the extremes of the TPCs by grouping the expression profile of samples according to temperature and to day or night. Discriminant analysis closely grouped samples from treatments from day and night at 17 °C in accordance with similar levels of respiration and calcification rates observed in these conditions, indicating that the low rates of photosynthesis in daytime treatments had little effect on gene expression profiles at low temperature. Furthermore, samples from nighttime 32 °C were grouped near the 17 °C groups on all three discriminant factor (DF) axes, consistent with the fact that similar values of nighttime calcification and respiration rates were observed at 32 °C and 17 °C. By contrast, discriminant analysis defined separate groups for daytime and nighttime samples at 32 °C on DF1, corresponding with higher rates of calcification and respiration in the day versus the night. This suggests that photosynthesis influenced gene expression profiles at 32 °C in accordance with the positive effect of photosynthesis on calcification and respiration rates. Additionally, discriminant analysis distinguished separate groups for daytime and nighttime samples at 25 °C, which further implies that photosynthesis was important in influencing the pattern of gene expression observed here. Physiological phenotypes are not always easy to predict at the level of gene expression^67,68^, because mRNA levels do not necessarily reflect the expression, localization, activity or behaviour of the proteins they code for. It can also be difficult to assign a gene expression profile to a particular physiological response due to variation among individuals. Work on individual variation in physiological and molecular responses in microcolonies is an avenue of future research. Nevertheless, here we observed an alignment of global patterns of gene expression with patterns of physiological parameters, suggesting a high degree of coherence between the thermal response of *S.pistillata* at the physiological level and the level of the expression of ion transport genes.

### Extremes of the thermal performance curves and dissolved inorganic carbon transport

Patterns determined by three-way ANOVA and posthoc analysis also corresponded with the physiological responses observed in the TPCs. The down-regulation of a small core group of genes relative to 25 °C at both 17 °C and 32 °C, in the day and night, occurred in parallel with decreases in rates of calcification, and other physiological responses at the extremes of the TPC. Notably, this core group was made up of a set of genes linked to DIC transport and interconversion. Transport of DIC and its interconversion between CO_2_ and HCO_3_^−^ facilitated by carbonic anhydrases is essential for calcification in which DIC of both seawater and metabolic origin is transported from the calicoblastic cells to the ECM^33,38^. Equally, DIC is vital for symbiont photosynthesis for which DIC from the surrounding seawater is transported to symbionts across the oral ectoderm and into the symbiont-containing endoderm, where it is ultimately used in the form of CO_2_^38,69^. Here we observed the downregulation at high and low temperatures in day and night of the four forms of carbonic anhydrase analyzed here (SpisCA1-4). Three of these CAs have been previously localized, including the SpisCA1 gene which is localized to the calcifying calicoblastic cell layer^40^, and SpisCA2 and SpisCA3 which are present in oral endoderm and aboral tissue (including the calicoblastic layer)^70,71^. Additionally, SpisCA2 protein has also been shown to be incorporated in the coral skeleton^72^. The core group also included representatives of two families of bicarbonate transporters SpisSLC26β and SpisSLC4γ^41^ for which there is also localization data from previous work. The bicarbonate transporter SpisSLC4γ has also been localized to the calicoblastic cells and is known to be specific to calcifying cnidarians^41^, and SpisSLC26β appears to be expressed ubiquitously in coral tissues including the calcifying cells^41^.

Overall our results are consistent with several previous studies on thermal stress in corals which have also observed downregulation of DIC-related genes at elevated temperature, including carbonic anhydrases in various coral species^13,17,18,24^ and a SLC26 gene in the coral *Porites astreoides*^17^. This previous work on thermal stress observed downregulation of DIC transport related genes solely at elevated temperature without taking into account the interaction of light. Here, our study shows that downregulation of genes involved in DIC transport associated with declines in calcification occurs at both low and high temperatures and also in day and nighttime conditions. Our results suggest that temperature-driven downregulation of DIC-related genes is largely independent of photosynthesis (which displayed low rates at 17 °C and was absent at night). Instead, our findings suggest that this response is associated with depressed rates of respiration which occur at both cold and warm ends of the TPC. Considering that several of the genes we analysed are localized to the calicoblastic layer (e.g. SpisSLC4γ), and that a large proportion of DIC used in calcification comes from metabolism^33,38^, our results may indicate disruption of the supply of DIC from respiration in the calcifying cells to the ECM. Further research is required to explore this possibility.

### Gene expression associated with an interaction of temperature and daytime conditions

A more diverse group of genes was associated with elevated temperature (32 °C) and daytime conditions and coincided with increased daytime calcification and respiration rates relative to the night and relative to 17 °C. These genes did not respond to day and night at 17 °C. This group included the upregulation of genes from all three ion transport process considered here i.e. DIC transport (e.g. SpisSLC26α, SpisSLC26β and SpisCA2 and SpisCA4), calcium transport and pH regulation (e.g. PMCA3 and the sodium proton exchanger NHE). It was notable that the carbonic anhydrase genes SpisCA2 and SpisCA4 were upregulated in the light, as previous investigations have also observed upregulation of carbonic anhydrases associated with light enhanced calcification^73,74^.

Overall this diverse group of genes suggests DIC, proton and calcium transport are all involved in a complex response specifically linked to elevated temperature stress and its interaction with light. Indeed, these genes may be linked to processes that are involved in keeping light calcification at higher rates at elevated temperature 32 °C than at 17 °C. This raises the possibility that at either extremes of the TPC the carbonate chemistry of the ECM is distinctly different. This possibility is supported by previous work on isotope signatures in skeletal chemistry, which indicates that increases in temperature can affect ECM chemistry through changes in both pH and DIC concentration^35,75^.

### Consequences of bleaching for calcification and ion transport

In the daytime at 32 °C, the photosynthetic rates per cm^2^ of coral colony were shaped simultaneously by a loss of symbiotic dinoflagellates together with thermally-driven increase in photosynthetic oxygen production rates. This runs contrary to the idea that mechanisms of bleaching at elevated temperature frequently begin with declines in symbiont photosynthetic efficiency^7,76^, but this observation is not inconsistent with previous research, as bleaching has also been suggested to begin with cellular dysfunction at the level of the mitochondria in the coral host^14,77^. Furthermore, in certain cases symbiotic dinoflagellates that have been expelled from the coral host during bleaching, can still be photosynthetically competent, indicating that bleaching may occur through dysfunction of the symbiosis rather than the dinoflagellates themselves^78^. In the context of the current study, we were interested in what this meant for calcification and its associated ion transport. Regardless of whether photosynthetic rates changed *per se* or by decreases in symbiont density, it may have amounted to a similar effect on ion transport for calcification. Indeed, a lower photosynthetic rate per unit of surface area of coral colony may have resulted in less of a pH increase in the coelenteron with a less favorable gradient for pH regulation of the ECM, together with less energy for calcification^50,52,79^. On the other hand, we cannot exclude the possibility that at both higher and lower temperatures the bleaching process may have involved cellular stress mechanisms including oxidative damage^80–82^, that impaired the molecular machinery involved in calcification^14^. Another possibility is that loss of algal symbionts could have reduced the supply of organic matrix precursor compounds that are known to be produced by the algae and are important in calcification^83^. Further research is needed to investigate these possibilities.

### Conclusions and perspectives for future research

Recent years have seen a succession of thermal stress events that underscore the vulnerability of corals and coral reef ecosystems to climate change^84^. In response, the field of coral research has begun to more actively explore interventions that could be used to enhance the physiological resilience of corals and their capacity to withstand environmental change. Approaches including assisted gene flow between coral populations, assisted evolution and synthetic biology are receiving attention as possible means to manipulate coral physiology to enhance coral survivorship and reproductive success in a rapidly changing ocean^20,21,85^. Putting aside the feasibly and risks of these approaches, their development would benefit from a deeper and more comprehensive understanding of coral physiology at molecular, cellular and organismal levels, and studies such as the current one are becoming more valuable in this context. Rigorously controlled experimental conditions like those used here, do not simulate natural environmental thermal stress, but instead they exclude the covariation of multiple environmental parameters allowing mechanisms to be deciphered that would otherwise be masked by physiological noise. Furthermore, mechanistic studies such as the current one that attempt to align patterns of gene expression with physiology, may highlight certain molecular pathways linked to coral resilience that could later become targets for further investigations using gene editing techniques such as CRISPR/Cas9^85,86^.

The current study was limited to a small set of genes linked to ion transport and in reality these genes operate in the context of a network interacting with higher levels of biological organization that we could not account for. Furthermore, we limited our study to coral host genes and did not consider molecular responses of the symbiotic dinoflagellates, which would be an interesting avenue for future research. Despite these limitations, we observed a remarkable degree of coherence with the global patterns of ion transporter gene expression with the physiological response of *S. pistillata*, suggesting that ion transport mechanisms are involved in the thermal sensitivity of this coral. More specifically, we observed a core response of a set of genes linked to DIC transport under conditions of declines in calcification at both high and low temperatures, concomitant with changes in respiration. Secondly, we observed that the combined effects of elevated temperature and light on calcification coincided with the differential expression of genes linked to calcium, H^+^ in addition to DIC transport. Further research needs to be carried out to understand the significance of these observations. One possibility to investigate is that the temperature and light-driven gene expression profiles we observed here point to changes in ion transport to the extracellular calcifying medium (ECM), which could result in changes in ECM composition with knock-on effects to calcification rate. This possibility is supported by previous geochemical investigations of the coral skeleton which indicate that temperature may be an important factor influencing ECM composition^75^. Follow-up work to the current study could employ recently developed *in vivo* approaches^30,34,87^ to characterize the effects of temperature on pH, and DIC and calcium concentrations in the ECM in living corals. Research of this kind may prove fruitful for a better understanding of the vulnerability and resilience of corals facing a rapidly changing ocean environment.

## Supplementary information

Supplementary Information
