# Supplementary material for: Ion transporter gene expression is linked to the thermal sensitivity of calcification in the reef coral Stylophora pistillata
Source: Sci Rep. 2019 Dec 10;9:18676. doi: 10.1038/s41598-019-54814-7 (PMC6904480; doi:10.1038/s41598-019-54814-7)
Supplement: Supplementary file 1 — Supplementary Information [file 41598_2019_54814_MOESM1_ESM.pdf]

Supplementary information

Ion transporter gene expression is linked to the thermal sensitivity of calcification in the reef coral *Stylophora pistillata*

Bernardet C[1,2], Tambutté E[1], Techer N[1], Tambutté S[1] and Venn AA[1]*

1. Centre Scientifique de Monaco, Marine Biology Department, 8 Quai Antoine 1er, Monaco 98000

2. Sorbonne Université, Collège Doctoral, F-75005 Paris, France

***Corresponding authors email: avenn@centrescientifique.mc**

Telephone: + 377 97 77 44 72

Supplementary Table 1. List of *S. pistillata* ion transport genes with RT-PCR primers and product size. Further information can be obtained from the Cnidarian Database www.data.centrescientifique.mc (Karako Lampert et al. 2014).

Target gene	Reference	Real-time PCR primers		Product size
Ca^{2+} channel	Zoccola *et al.* (1999)	F	TGCTACAAGATCGCCACCAACAC	82 pb
		R	TCAGCCGCCAGCAGAATACTACTC	
NHE	Capasso *et al.* (in prep)	F	TCATTCTCCTTGGCTGTTTTACTG	138 pb
		R	CATGAAGAATCCGCACAAGAGGTT	
PMCA1	Zoccola *et al.* (2004)	F	CCAAAACCTCCCAAAACCTTCCT	131 pb
		R	GACCCCTCGCAATCCTCTGG	
PMCA2	Wang *et al.* (in prep)	F	GTACTGGCAAGAGCAACACC	125 pb
		R	AAGACTGGCCCATCATTAGC	
PMCA3		F	TCACTGGAGATCCGCGTTTTTACA	140 pb
		R	ACCGGTTTGCTTTGGGAGACATT	
SpisSLC4β	Zoccola *et al.* (2015)	F	CCAATGTTGCGTTCGGTAGTCTTT	143 pb
		R	GCCCTGTAGCGCCAATAATAATCA	
SpisSLC4γ		F	CTTGGAGGGCTGTTTCTTGAT	152 pb
		R	TATTTTCCGCTCCCTACCCTGTTC	
SpisSLC4δ		F	TCTTGCTGGTGGAATTGGTGGAGT	150 pb
		R	TAAACGGAATGGCATGTAGTCAAG	
SpisSLC4ε		F	GGCTGCCTACCCACCAAACCA	113 pb
		R	GGGGCAAATCCAAATCCACATAG	
SpisSLC26α		F	GCGAAATTTCCTGCCATCTA	133 pb
		R	TCGCCACGGTTATCCCTCCTGCTA	
SpisSLC26β		F	CATCGCGGCACTTGTCTGTAG	159 pb
		R	GTCCATAACGCGGTGCCTTCTGTC	
SpisSLC26γ		F	GATTGCCGCGTCATTTCACTG	131 pb
		R	ATTCCCTGCATTTCCCCTGTAA	
SpisCA1	Moya *et al.* (2008)	F	GGAGGGACCCGACACGTGGAA	110 pb
		R	AGTCGGCAAGTCCCGGTTCG	
SpisCA2	Bertucci *et al.* (2011)	F	TGCGGCCGAGTGACTGGAGA	91 pb
		R	CGCCACTGAGTTTAGCGCCTCC	
SpisCA3	Del Prete *et al.* (2018)	F	TGGCTCTGGTGGCCCAAATGGT	136 pb
		R	TGCTGGGCACGGTGACAGAGGT	
SpisCA4	Le Goff et al. 2016	F	GGTGCCGCTTGTTTTCAGCGCA	87 pb
		R	TCCGGTTGCGCATGTCCCTGT	
V-ATPase	Capasso *et al.* (in prep)	F	GAGGAGTTTGATTTTGGGGAAGTA	97 pb
		R	AGTCGCAAATAAGAGGCTGTGTT	
L22	Karako Lampert et al. 2014	F	TGATGTGTCCATTGATCGTC	137 pb
		R	CATAGGTAGCTTGTGCAGATG	
36B4	Moya et al., 2008	F	AACAAGGTGGCAGCCCCAGC	91 pb
		R	GTCTTCTCGGGACCCAGGCCA	

Supplementary Table 2. Results of two-way ANOVA tests on the effect of temperature and day/night on calcification and respiration rates (Figure 1A and B) and the results of one-way ANOVA tests on the effect of temperature on photosynthetic rate (Figure 1C) and symbiont density (Figure 1D).

Variable	Effect	df	F	P	Post-hoc
Calcification rate	Temperature	9, 118	70.203	< 0.00001	See table S3
	Time	1, 118	285.72	< 0.00001	See table S3
	Temperature x Time	9, 118	7.386	< 0.00001	See table S3
Respiration rate	Temperature	9, 124	36.381	< 0.00001	See table S4
	Time	1, 124	115.537	< 0.00001	See table S4
	Temperature x Time	9, 124	2.292	< 0.05	See table S4
Photosynthetic rate	Temperature	9, 62	18.72	< 0.00001	21,23,25,27,29,31 > 21,27,19,32 > 17,33
Symbiont density	Temperature	4, 10	11.14	< 0.001	25°C ≥ 31°C ≥ 19°C > 17°C, 32°C

Supplementary Table 3. Results of post-hoc pairwise t tests on calcification rate. Letters (x and y) indicate statistical differences (p < 0.05) with respect to 25 °C, and between night and day.

Temperature	Time	25°C		Difference between night / day
		Night	Day	
17°C	Night	x		-
	Day		x	
19°C	Night	-		-
	Day		x	
21°C	Night	-		y
	Day		x	
23°C	Night	-		y
	Day		x	
27°C	Night	-		y
	Day		-	
29°C	Night	-		y
	Day		x	
31°C	Night	-		y
	Day		-	
32°C	Night	x		y

	Day		x	
33°C	Night	x		y
	Day		x	

Supplementary Table 4. Results of post-hoc pairwise t tests on respiration rate. Letters (x and y) indicate statistical differences ($p < 0.05$) with respect to 25 °C, and between night and day.

Temperature	Time	25°C		Difference between night / day
		Night	Day	
17°C	Night	x		-
	Day		x	
19°C	Night	x		-
	Day		x	
21°C	Night	x		y
	Day		x	
23°C	Night	x		y
	Day		-	
27°C	Night	-		y
	Day		-	
29°C	Night	-		y
	Day		-	
31°C	Night	-		y
	Day		-	
32°C	Night	x		y
	Day		x	
33°C	Night	x		y
	Day		x	

Supplementary Table 5. Eigen values from descriptive discriminant analysis of transcellular transporter genes

	DF1	DF2	DF3	DF4	DF5
Eigen values	0.967	0.95	0.905	0.776	0.419
Proportion	**24.081**	**23.638**	**22.536**	19.313	10.434
Cumulative Proportion	**24.081**	**47.718**	**70.254**	89.566	100

Supplementary Table 6. Discriminant variables from descriptive discriminant analysis of transcellular transporter genes

	DF1	DF2	DF3	DF4	DF5
Ca^{2+} channel	-1.25174	-0.50275	0.82883	0.92	1.50667
PMCA1	1.49177	2.30532	-2.37027	0.7289	-3.0821
PMCA2	0.84803	0.53783	2.229	-2.0158	-4.70668
PMCA3	-0.412	0.39785	1.07867	-0.6077	1.01399
NHE	-2.04806	-1.61509	-0.80468	-1.4671	-0.78038
SpisSLC4β	0.13748	0.39	-0.57168	1.2192	-3.02595
SpisSLC4g	-0.1545	-0.51738	0.27089	3.0924	1.27347
SpisSLC4γ	0.55733	0.26041	0.22834	-1.1867	-1.45829
SpisSLC4e	0.61186	-0.91709	0.16733	-1.0479	4.7902
SpisSLC4δ	0.14967	-0.02118	-1.00771	1.0861	-1.40355
SpisSLC26b	-0.02647	0.06913	-0.23149	0.1728	0.12823
SpisSLC4ε	-0.41848	-0.03035	0.69804	0.9642	-0.71527
SpisCA1	-0.58458	-0.50437	1.3165	-2.7138	-0.08673
SpisCA2	0.19726	0.62296	-0.3189	0.2463	-1.87507
SpisCA3	0.77479	0.93835	-1.31863	1.9029	0.03303
SpisCA4	-1.0053	-0.57662	0.2773	-1.0983	1.76961
V-ATPase	0.23082	-1.08646	0.07618	-1.0984	6.54858

Supplementary Table 7. Results of three-way ANOVA tests on the effect of temperature (17, 25 and 32ºC), time of day (day/nighttime) and gene identity on relative gene expressions (Figures 3 and 4 in main mansucript). Results of posthoc tests are given in main manuscript in Tables 1 and 2.

Variable	Effect	df	F	P
gene expression	Temperature	2, 510	85.8017	< 0,00001
	Time	1, 510	46.8169	< 0,00001
	Genes	16, 510	4.1862	< 0,00001
	Temperature*Time	2, 510	12.3359	< 0,00001
	Temperature*Genes	32, 510	16.9948	< 0,00001
	Time*Genes	16, 510	4.0744	< 0,00001
	Temperature*Time*Genes	32, 510	2.8592	< 0,00001

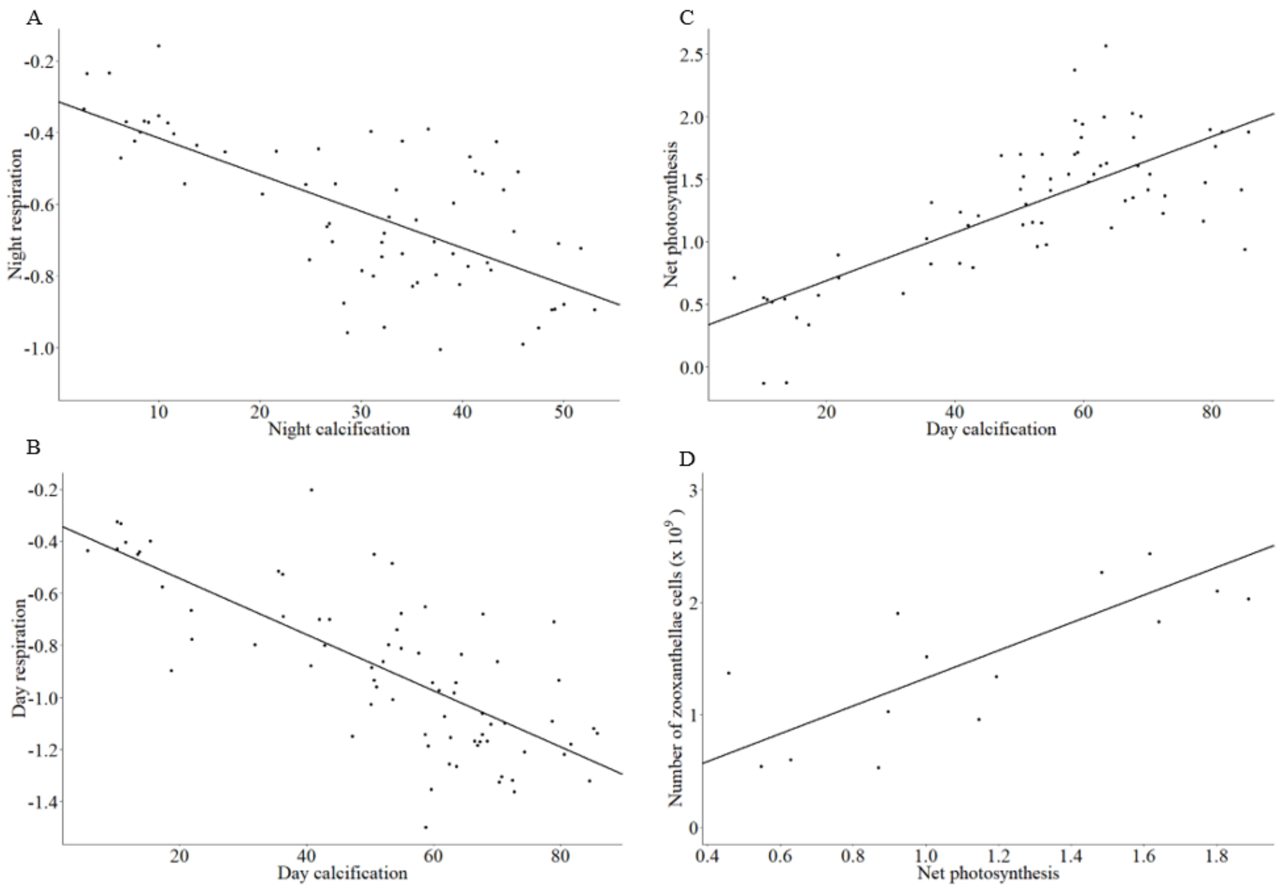

Supplementary Figure 1. Correlation of (A) night respiration to night calcification ($p = 1.6\ 10^{-10}$ and $R^2 = 0.47$), (B) day respiration to day calcification ($p = 3.1\ 10^{-14}$ and $R^2 = 0.56$), (C) net photosynthesis to day calcification ($p = 3.1\ 10^{-13}$ and $R^2 = 0.56$), (D) symbiont density to net photosynthesis ($p = 4.4\ 10^{-4}$ and $R^2 = 0.60$).

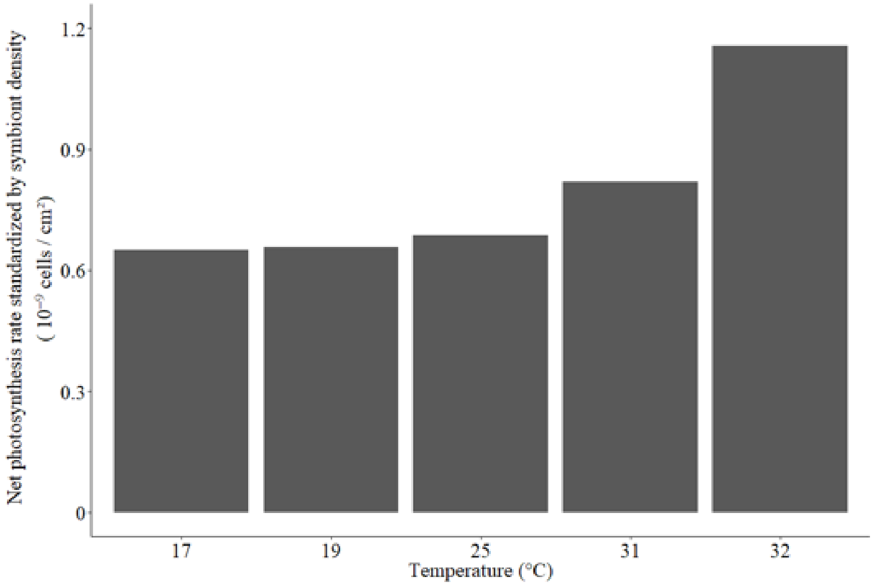

Supplementary Figure 2. Photosynthetic rate normalized to symbiont density.

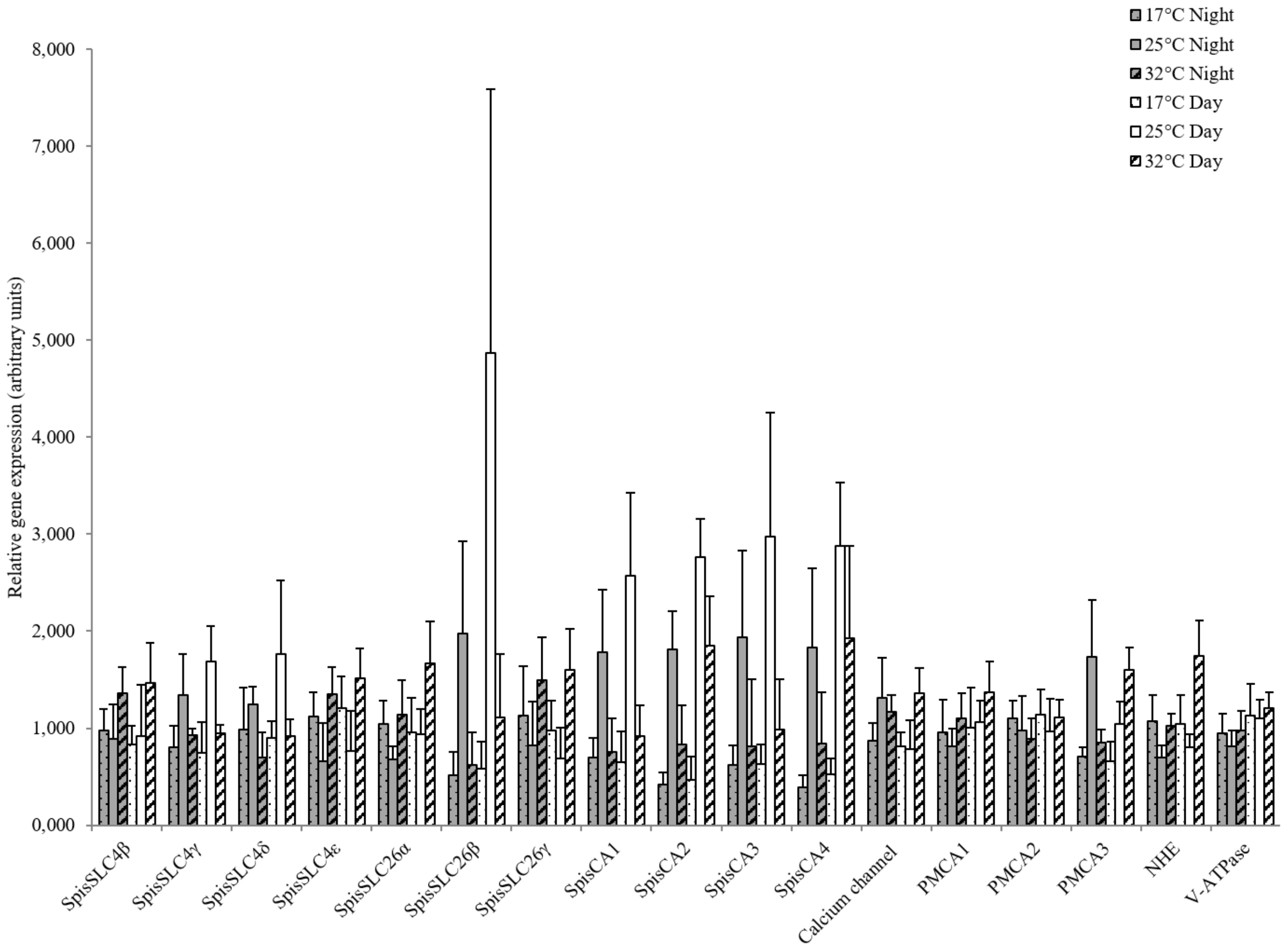

Supplementary Figure 3. Gene expression (mean ±SD) of ion transport genes relative to geometric mean of expression of the reference genes L22 and 36B4. The same data is presented in the main manuscript as log10 transformation plots in Figure 3 and 4 relative to 25ºC and nighttime.

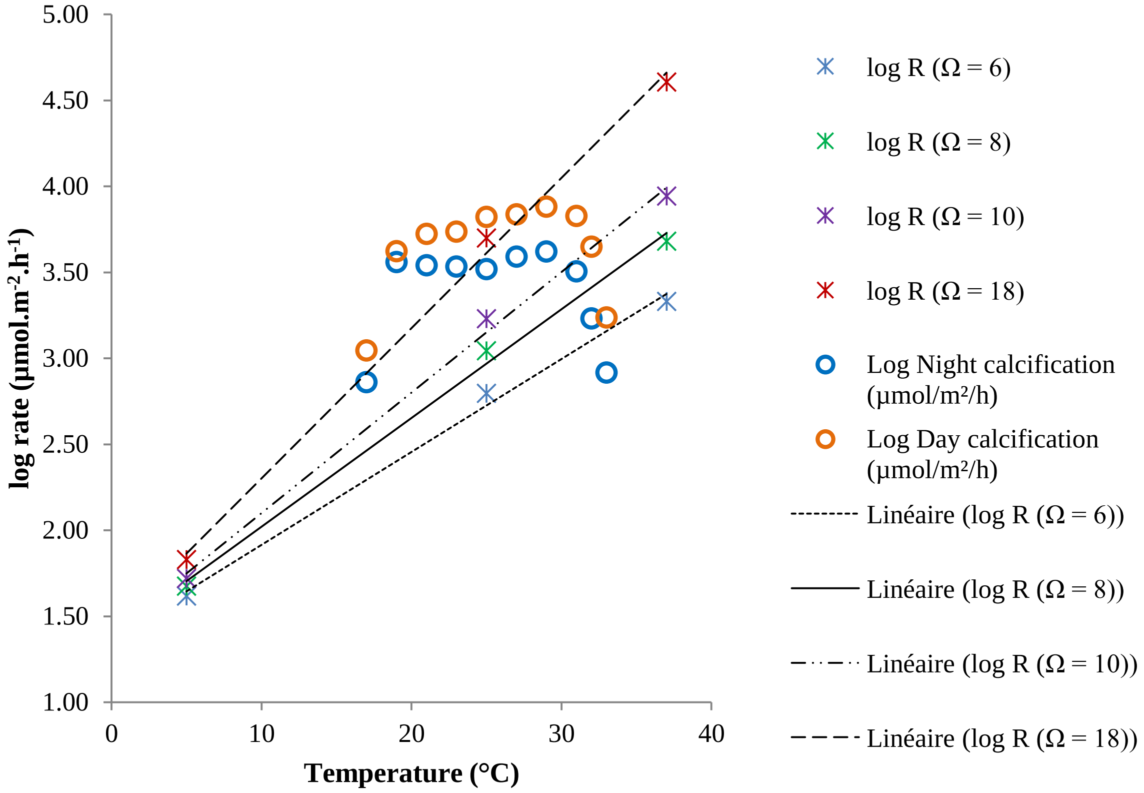

Supplementary Figure 4. Abiotic precipitation rate of aragonite as a function of aragonite saturation state at 5°C, 25°C and 37°C in seawater (modified from Burton and Walter, 1987), and precipitation rate of aragonite in *S. pistillata* from 17°C to 33°C during night (blue circles) and day (orange circles).

References

Bertucci A, Tambutté S, Supuran CT, Allemand D, Zoccola D (2011) A New Coral Carbonic Anhydrase in Stylophora pistillata. *Marine Biotechnology*, **13**, 992–1002.

Del Prete S, Bua S, Zoccola D et al. (2018) Comparison of the Anion Inhibition Profiles of the α-CA Isoforms (SpiCA1, SpiCA2 and SpiCA3) from the Scleractinian Coral Stylophora pistillata. *International Journal of Molecular Sciences*, **19**, 2128.

Le Goff C, Ganot P, Zoccola D, Caminiti-Segonds N., Allemand D, and Tambutté S. (2016). Carbonic Anhydrases in Cnidarians: Novel Perspectives from the Octocorallian Corallium rubrum. PloS ONE 11(8): e0160368. https://doi.org/10.1371/journal.pone.0160368

Karako-Lampert S, Zoccola D, Salmon-Divon M et al. (2014) Transcriptome Analysis of the Scleractinian Coral Stylophora pistillata (ed Voolstra CR). *PLoS ONE*, **9**, e88615.

Laurent J1, Venn A, Tambutté É, Ganot P, Allemand D, Tambutté S. Regulation of intracellular pH in cnidarians: response to acidosis in Anemonia viridis. FEBS J. 2014 Feb;281(3):683-95. doi: 10.1111/febs.12614. Epub 2013 Dec 10.

Moya A, Tambutté S, Bertucci A et al. (2008) Carbonic anhydrase in the scleractinian coral Stylophora pistillata: Characterization, localization, and role in biomineralization. *Journal of Biological Chemistry*.

Zoccola D, Tambutté E, Kulhanek E, Puverel S, Scimeca JC, Allemand D, Tambutté S (2004) Molecular cloning and localization of a PMCA P-type calcium ATPase from the coral Stylophora pistillata. *Biochimica et Biophysica Acta - Biomembranes*.

Zoccola D, Ganot P, Bertucci A et al. (2015) Bicarbonate transporters in corals point towards a key step in the evolution of cnidarian calcification. *Scientific Reports*, **5**, 9983.

Zoccola D, Tambutté E, Sénégas-Balas F, Michiels J-F, Failla J-P, Jaubert J, Allemand D (1999) Cloning of a calcium channel α1 subunit from the reef-building coral, Stylophora pistillata. *Gene*, **227**, 157–167.
